# Reporting a Case of Solid Pseudopapillary Neoplasm of the Pancreas in a 44-Year-Old Woman with Parallel Analysis of Literature

**DOI:** 10.1155/2023/1768926

**Published:** 2023-04-28

**Authors:** Sargun Singh, Qing Zhao, Teviah E. Sachs, Kevan Hartshorn

**Affiliations:** ^1^Division of Gastroenterology and Liver Disease, Case Western Reserve University School of Medicine, Cleveland, OH, USA; ^2^Department of Pathology, Boston University School of Medicine, Boston, MA, USA; ^3^Section of Surgical Oncology, Department of Surgery, Boston University School of Medicine, Boston, MA, USA; ^4^Section of Hematology Oncology, Department of Medicine, Boston University School of Medicine, Boston, MA, USA

## Abstract

We present a distinctive case of solid pseudopapillary neoplasm as seen in a 44-year-old woman who presented with an abdominal mass but unremarkable labs with no elevation in any of the tumor markers. Her symptomatology ranged from typical symptoms suggestive of malignancy such as weight loss, lethargy, and anorexia to complaints like abdominal pain and jaundice. Prior to presenting at our center, she was given no hope or much in terms of treatment options. She was found to have a substantial mass over the body and tail of pancreas with characteristic and typical gross as well as histological features. Subsequently, she underwent a successful surgery and has found herself in remission since.

## 1. Introduction

Solid pseudopapillary tumor or solid pseudopapillary neoplasm of pancreas (SPN) is a rare entity. Its unique characteristics include occurrence in young females, uncertain histogenesis, borderline malignant potential, and excellent long-term prognosis, even when the initial presentation is aggressive. Its etiology and pathogenesis remain uncertain warranting discussion as more cases come to the fore due to the recent advancements in imaging and an increase in overall awareness and curiosity surrounding this tumor. Histopathological analysis remains the gold standard to reach a definitive diagnosis. Herein, we discuss the case of a 44-year-old woman who presented with an abdominal mass and was subsequently diagnosed with SPN.

## 2. Case Report

**Figure 1:**
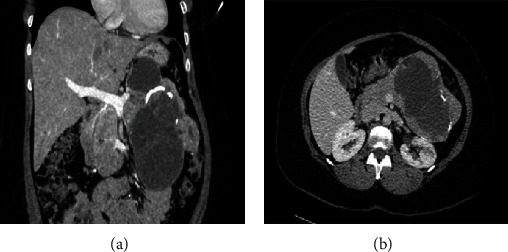
(a) Large solid-cystic mass with calcifications, distorting pancreas. (b) Large mass originating from the body and tail of the pancreas abutting the left kidney and spleen.

A 44-year-old obese woman, with no significant past medical history and otherwise in good health, presented with a left-sided abdominal mass that she had had for the past one year. She denied having experienced abdominal pain, nausea, vomiting, fullness, diarrhea, flushing, weight loss, anorexia, fatigue, jaundice, fever, or chills. She had undergone an exploratory laparotomy in Haiti a month earlier which was aborted. She was given the diagnosis of pancreatic cancer and told that there was little that could be done with respect to treatment. On physical examination, a mass was palpable in the left upper quadrant of her abdomen. Complete blood count and chemistry panels (including electrolytes and liver function tests) were in the normal range, except for a slightly elevated fasting blood glucose level of 103 mg/dl. The tumor markers were within the normal range with a carcinoembryonic antigen (CEA) level of 0.9 ng/ml and carbohydrate antigen 19-9 (CA 19-9) level of 5 U/ml. Computerized tomography (Figures 1(a) and 1(b)) demonstrated a very large mass with solid and cystic features in addition to calcifications originating from the body and tail of the pancreas which was grossly distorted and irregular.

The mass impinged on the neighboring structures including the stomach and duodenum and abutted the left kidney and spleen. The mass measured approximately 13 by 13 cm in its greatest dimensions. There was neither any evidence of a disseminated disease nor of direct local invasion. Irregular heterogeneous enhancement of the irregularly thickened walls of multiple cysts and of the solid components of the mass was noted. No evidence of ascitic fluid or bowel obstruction was found, and no lymphadenopathy was observed. The case was discussed at a multidisciplinary tumor board, and the consensus was to proceed with resection without biopsy as further tissue sampling would not alter management but would risk tumor rupture and peritoneal dissemination.

The patient was operated based on a presumptive diagnosis of SPN of the pancreas. She underwent an exploratory laparotomy with distal pancreatectomy and en-bloc splenectomy with extended left colectomy and primary colo-colonic anastomosis.

**Figure 2:**
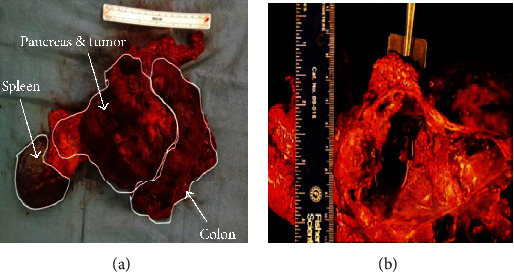
(a) 15.5 cm pancreatic mass attached to the colon and intact spleen. (b) Encapsulated cystic, hemorrhagic, and solid mass in the tail of the pancreas.

Findings on gross examination (Figure 2(a)) showed a large, encapsulated mass of approximately 15 cm occupying the tail and body of the pancreas that was adherent to the splenic flexure of the colon. The tumor was serially sectioned (Figure 2(b)) to reveal cystic and solid cut surface with focal necrosis, calcifications, and cysts containing bloody fluid.

**Figure 3:**
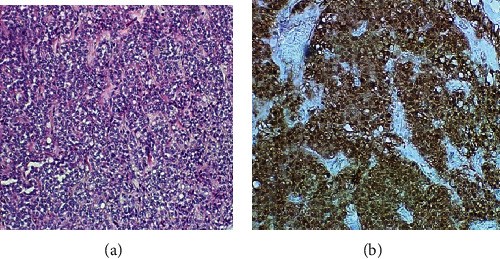
(a) Monomorphic cells with solid and pseudopapillary architecture admixed with hyalinized to myxoid stroma. (b) Immunochemical stain for *β*-catenin nuclear was strongly positive. The *β*-catenin staining was done using a Ventana Benchmark Ultra Instrument and the Ventana Optiview DAB staining kit. The antibody was Clone 14 from Ventana Medical Systems (Oro Valley, Arizona, USA).

On microscopic examination (Figure 3(a)), the tumor was found to be composed of monomorphic cells with solid and pseudopapillary architecture mixed with hyalinized to myxoid stroma with degeneration and dystrophic calcification. Some of the tumor cells had eosinophilic or clear cytoplasm. Necrosis or high-nuclear-grade atypia was not seen, though mitotic figures were present.

Immunohistochemistry revealed the tumor to be positive for nuclear *β*-catenin (Figure 3(b)) and low molecular keratin Cam 5.2. It was focally positive for progesterone receptor (PR) but negative for synaptophysin, chromogranin, and estrogen receptor (ER) (data not shown). The Cam 5.2, PR, ER, synaptophysin, and chromogranin antibodies were also obtained from Ventana and developed with the same system as the *β*-catenin.

The patient was discharged after five days in a good condition. No adjuvant therapy was given. The postoperative recovery was significant for a mild pancreatic leak, which was controlled with drainage. The patient has been followed up for over four years without any evidence of recurrence. The table (Table 1) summarizes the findings specific to our case; these are classic for SPN.

The above findings together with the clinical picture confirmed the diagnosis.

## 3. Discussion

SPN is a rare exocrine tumor of the pancreas with low malignant potential and a reasonably good prognosis [1], rarely recurring postexcision [2, 3]. Also known as Frantz's tumor, having been described for the first time, in 1959, by Dr. Virginia Kneeland Frantz as a papillary-cystic tumor of the pancreas in a 2-year-old male patient, it was included in the WHO classification in 1996 [4]. Although rare in its occurrence, more and more cases are being brought to light because of the advancements in imaging modalities. In a review by Law et al. [5], 2744 patients of SPN were identified with over 87% being reported post 2000, marking a seven-fold increase in the reporting of cases between 2000 and 2012 when compared to the period from 1961 to 1999 with men contributing to 12.2% of the cases. This is not necessarily representative of increased incidence but rather of increased awareness and better means of diagnosing. The tumor can form large masses as evident in our case report where the size of the excised mass exceeded 15 cm. The review by Papavramidis and Papavramidis [6] analyzed 718 reported cases of SPN in the English literature at the time. The mean diameter of the tumor was observed to be around 6.08 cm with the overall range being between 0.5 and 34.5 cm. They further highlighted the predilection of the tumor for the female gender with a female to male ratio of 9.78 : 1. The most common location remains the tail and the body of the pancreas [7]. The patients mostly fall into the second or third decades of their lives though our patient was past the age of forty when she presented. There are reported cases of SPN in children [8], older adults, and men [9] as well.

The pseudopapillary pattern [10] results from the fact that these tumors begin as solid masses and have many unsupported tiny blood vessels. The cells which are farthest from the vessels undergo necrosis due to nutritional deprivation whereas cells next to the vessels stay intact resulting in characteristic cystic changes and formation of the typical pseudopapillary pattern associated with the tumor. Hence, cystic changes are not the norm in SPN and are mostly seen in larger masses that have undergone necrosis and degenerative changes having outgrown their blood supply [2, 11]. However, these characteristic pseudopapillary features alternating with solid areas are important to make the tissue diagnosis [12, 13]. The SPN cells are known to express beta-catenin, E-cadherin, vimentin, alpha-1 antitrypsin and alpha-1 chymotrypsin, and CD10 and CD56 and are negative for pancreatic enzymes and chromogranin [10, 13]. A 100% of SPNs have been found to have beta-catenin pathway mutations, specifically mutations of the CTNNB gene [14] that can be detected in fine needle aspirates of the tumors [15]. The labs are mostly insignificant with no rise in the amylase or lipase levels and without the elevation of tumor marker levels, a finding usually associated with pancreatic carcinomas. In a single-institution study by Beltrame et al. [16] comprising 451 patients with cystic tumors of the pancreas, 18 (3.7%) were found to have SPN of the pancreas through histological analysis and only one patient was noted to have an elevated serum CA 19-9 level of 92 U/ml (normal range being 0 to 37 U/ml). Beta-catenin was always expressed in these patients as well. The physical examination is generally not of much use either other than the finding of a palpable large mass. The patients can range from being asymptomatic to having unclear and vague symptoms like abdominal discomfort and pain or compression symptoms when the extensive mass impinges upon the neighboring structures [13]. Most tumors are diagnosed as incidental imaging findings. Surgical excision or enucleation of the neoplasm is almost always curative [17, 18]. Metastasis is rare and if present is not a contraindication to surgical removal [19]. Resection of metastases along with or after resection of the primary is still compatible with long-term disease-free survival. The most common site of metastasis is the liver [12]. Perineural invasion, angioinvasion, and invading of neighboring structures and tissues along with features such as large size, cellular or nuclear atypia, and high mitotic rate have been associated with increased malignant potential and a higher rate of recurrence of SPNs [16]. In terms of imaging, focal discontinuity of the capsule is suggestive of malignant potential as per a study by Huang et al. [20]. Distal pancreatectomy with or without splenectomy is done for the tumors in the body and tail of the pancreas. Duodenopancreatectomy is done for the tumors arising in the head of the pancreas [19]. Pancreatic fistula is the most common postoperative complication as noted in our case [4, 17, 18, 21].

Even though this neoplasm mostly favors the female gender, no gender-specific variations have been noted as such but some of these neoplasms have been reported to demonstrate positivity for progesterone receptors [22]. In our case too, the tumor was positive for the progesterone receptor and negative for the estrogen receptor. Machado et al. [23] observed that SPNs in male patients were more aggressive compared to the female gender. Cai et al. [24] studied 16 male patients with SPNs and observed that the male patients were older and had a more favorable outcome after surgery in comparison to females, with no recurrence or death in the follow-up period. In contrast, a recent population study by Wu et al. [25] revealed that male patients had significantly poorer overall and disease-specific survival when compared to their female counterparts. The same study noted more male cases of SPN beyond the age of 65 which could be a contributory factor to the poorer prognosis since older age is associated with an overall poor outcome. Recently, Law et al. [26] reported that addition of endoscopic ultrasound-guided fine needle aspiration (EUS-FNA) to a preoperative work-up of SPN increased the diagnostic yield to 82.4%. However, some refrain from FNA due to risk of peritoneal dissemination and associated complications. The diagnosis is confirmed based on the typical solid and cystic and pseudopapillary findings on microscopic examination.

The differential diagnosis of the SPNs can be challenging. Pancreatic endocrine neoplasms can mimic SPNs; however, the presence of pseudopapillary architecture in our case and the lack of endocrine (salt-and-pepper) chromatin favor SPN. Also, the beta-catenin expression along with negativity for chromogranin goes in favor of SPN [10]. Acinar cell carcinoma is yet another differential to be considered but this one in contrast to SPN shows a predilection for the male gender. Histologically, the cells have granular cytoplasm, and the growth pattern of the tumor is acinar, trabecular, or solid. With respect to markers, it does express pancreatic enzymes but usually does not express beta-catenin [27]. Compared with ductal adenocarcinoma of the pancreas, they have no ductal dilatation. Another differential is a pancreatic pseudocyst which is usually preceded with a history of pancreatitis and lacks the typical imaging and histological features associated with SPN.

One important question is predicting which patients may recur and what must be the appropriate follow-up after surgery. A study done by Serrano et al. [28] found that more often recurrence occurs 5 to 7 years after complete surgical resection if at all which seems to indicate that a >5-year clinical follow-up is necessary with routine imaging after resection of SPN, especially in high-risk patients which show features such as invasion of lymphatics and blood vessels, metastases, and perhaps invasion of the tumor capsule [29]. Another meta-analysis [30] found a recurrence rate of 2% postresection, identifying male patients and patients with positive lymph nodes, R1 margins, and lymphovascular invasion to be more at risk for recurrence.

## 4. Conclusion

The SPN remains a unique diagnosis with vague presenting symptoms and a poorly understood natural history of the disease. An enlarging abdominal mass is the most common presenting complaint, and surgery remains the mainstay treatment of choice. The etiology and the vulnerable predisposition of the female gender remain unclear warranting further research into understanding the mechanisms associated with the tumor. The prognosis is excellent with most patients showing no recurrence of the tumor postexcision. The rather perplexing nature of the neoplasm makes it a riveting case worthy of reporting.

## Figures and Tables

**Table 1:** Findings in our case that are typical for SPN.

Investigation	Findings
(1) CT scan	A large heterogeneous mass with solid as well as cystic features originating from the body and tail of the pancreas. Calcifications.
(2) Gross examination	An encapsulated large mass occupying the tail of the pancreas with solid components, focal necrosis, calcifications, and cysts filled with bloody fluid.
(3) Microscopic picture	Solid and pseudopapillary pattern noted along with degeneration and dystrophic calcification.
(4) Immunohistochemistry	Positive: beta-catenin, CAM 5.2, and progesterone receptor (PR)

## Data Availability

Data is available on the patient's medical records.
